# Delayed Gastric Leak Secondary to Marijuana Consumption Post Sleeve Gastrectomy

**DOI:** 10.7759/cureus.84999

**Published:** 2025-05-28

**Authors:** Sai Swarupa Vulasala, Sunil Sharma, Swati Sharma, Lourdhu Pragna R Onteddu, Smita Sharma

**Affiliations:** 1 Radiology, University of Florida College of Medicine – Jacksonville, Jacksonville, USA; 2 Surgery, New Life Surgical Associates, Jacksonville, USA; 3 Health Sciences, University of Ottawa, Ottawa, CAN

**Keywords:** bariatric surgery, diagnostic imaging, gastrectomy, obesity, substance-related disorders

## Abstract

Obesity is a chronic disease and a major health concern in the United States. Laparoscopic sleeve gastrectomy (LSG) is the most common metabolic and bariatric surgery performed in the United States. Complications after sleeve gastrectomy are rare and mostly observed during the acute phase. We present a case of delayed gastric leak after LSG secondary to marijuana consumption. In this case report, we discuss the clinical presentation, imaging characteristics and management of delayed gastric leak, and its association with marijuana consumption.

## Introduction

Obesity is a chronic disease characterized by pathophysiologic dysregulation with increased risk for multiple comorbid conditions such as asthma, low back pain, osteoarthritis, diabetes, dyslipidemia, cardiovascular diseases, gastroesophageal reflux diseases, and obstructive sleep apnea [1]. It is a major health concern in the United States, prevalent in every one in five children and two in five adults [2]. Metabolic and bariatric surgery (MBS) aids in the complete resolution or improvement of the majority of these comorbid conditions [3]. It also reduces the all-cause mortality rate compared to the non-surgical patients [4]. Based on the 2022 American Society for Metabolic and Bariatric Surgery (ASMBS) and International Federation for the Surgery of Obesity and Metabolic Disorders (IFSO), MBS is recommended in patients with body mass index (BMI) ≥ 35 kg/m^2^ irrespective of the comorbidities, and MBS should be considered in patients with BMI 30-34.9 kg/m^2^ and metabolic diseases [5]. Laparoscopic sleeve gastrectomy (LSG), Roux-en-Y gastric bypass, biliopancreatic diversion with duodenal switch, and loop duodenal switch with sleeve gastrectomy are the four major MBS types. LSG is the most common MBS and involves the removal of 75-80% of the gastric greater curvature and preserving lesser curvature and gastric pylorus [6]. Complications of sleeve gastrectomy include gastric leak, bleeding along the staple line, stenosis, marginal ulcer, and portal venous thrombosis [7-10]. Gastric leak is the most concerning complication along the proximal staple line, prevalent in around 0.5-1% of cases [7].

In this paper, we discuss a case of delayed gastric leak presented seven months after LSG secondary to marijuana consumption.

## Case presentation

A 30-year-old female with a BMI of 37.49 kg/m^2^ came to our clinic to pursue surgical management of her obesity. She has been suffering from chronic obesity for five to 10 years and has tried multiple weight loss remedies, such as dietary and lifestyle modifications. She also developed obesity-related comorbid conditions, including chronic back pain and hypertension. The patient denied a history of prior surgeries, smoking, or substance abuse. After a detailed discussion with the patient on the risks and benefits of MBS, a decision was made to proceed with LSG to aid with her medical comorbid conditions mentioned above. The surgery was successful without any perioperative complications.

**Figure 1:**
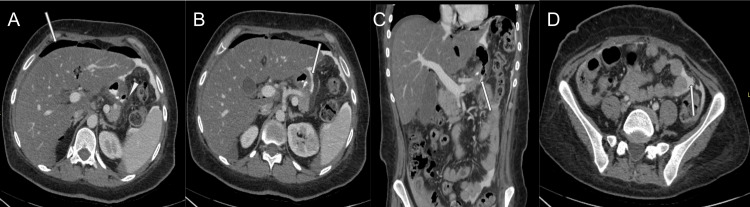
(A) Axial view of the computed tomography (CT) of the abdomen and pelvis demonstrating the gastric wall defect (white arrow head) with leakage of air into the peritoneum and associated pneumoperitoneum in the perihepatic space (white arrow). (B) Axial and (C) coronal CT of the abdomen and pelvis with oral contrast demonstrating contrast leak (white arrow) along the gastric wall defect into the peritoneal space. (D) Axial CT of the abdomen and pelvis demonstrating a leaked contrast material in the pelvis.

**Figure 2:**
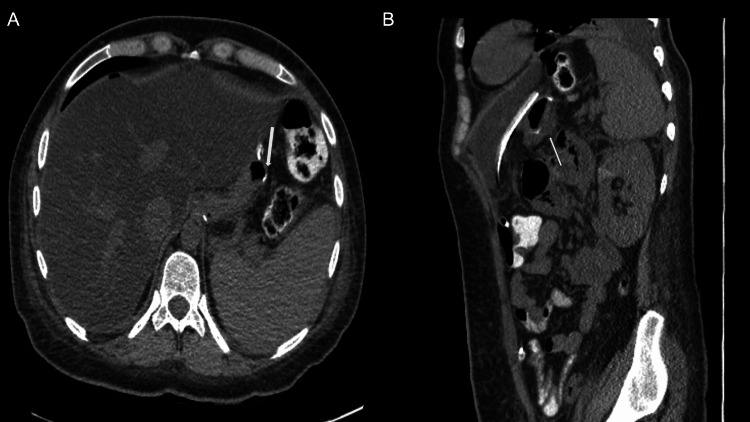
(A) Axial and (B) coronal view of the abdomen and pelvis demonstrating successful repair of the gastric leak (white arrows).

Approximately seven months after surgery, the patient presented with a one-day history of sudden, severe, sharp upper abdominal pain, which was associated with multiple non-bloody emesis. The patient denied diarrhea episodes, fever, or urinary symptoms. However, she endorsed using marijuana over the past month as she lost one of her family members. Computed tomography of the abdomen with intravenous and oral contrast demonstrated signs of gastric perforation (Figure 1) with oral contrast extravasation at the staple line and free intraperitoneal air. The patient was rushed to the operating room, where successful robotic-assisted repair of gastric sleeve perforation with abdominal washout was performed. Intraoperative findings include extensive gastric and biliary contents within the bilateral upper abdominal quadrants. Further exploration revealed a 2 cm perforation along the mid-portion of the stomach. The perforation site was further confirmed with intraoperative endoscopy. The perforation was sutured completely with an omental tuck (Figure 2). The closure was confirmed later to be watertight on a leak test using endoscopy. Postoperatively, the patient was asymptomatic, and her diet gradually advanced from a complete liquid to a solid diet. 

## Discussion

A gastric leak is the most serious complication and can be classified into acute, early, late, and chronic leaks if the presentation is within one week, one to six weeks, six to 12 weeks, and more than 12 weeks, respectively [10]. It is more common in the immediate postoperative period due to mechanical causes such as stapler misfiring or ischemic causes such as electrocautery dissection [11]. Distal gastric stenosis can impair gastric emptying, causing gastric distension and increased intraluminal mechanical pressure on the staple line [11]. Although the incidence ranges from 0.5% to 5%, gastric leak is associated with higher morbidity and mortality rates of up to 30% [7,12]. If chronic and untreated, the gastric leaks can progress to fistulas. Depending on their location, gastro-colic or esophagopleural-bronchial fistulas may develop [12]. 

Clinical symptoms of gastric leak may vary from asymptomatic to sepsis, multiorgan failure, and death. Upper gastrointestinal (UGI) fluoroscopy and computed tomography (CT) are reliable imaging modalities in identifying the location of the gastric leak, and the latter has the advantage of identifying additional complications such as peri-gastric collections or abscess formations. Leaks are more common along the proximal end of the staple line close to the gastroesophageal junction [13]. These manifest on the UGI fluoroscopy as extraluminal extravasation of water-soluble contrast material. If the leak is not identified with water-soluble contrast, then a high-density barium contrast can identify subtle leaks [13]. CT can help visualize the leaks not evident on UGI fluoroscopy and identify intra-abdominal fluid collections if present [14]. It is crucial to mention the location, extent, and severity of gastric leaks in the radiology report, which affects clinical management. The imaging findings can vary from phlegmonous changes, fluid collection with gas locules, contrast leak into the collection, and contrast leak into intraperitoneal space.

In our case report, the patient developed a gastric leak after a month of marijuana use. The temporal relationship between the onset of the gastric leak and the patient's initiation of marijuana use led us to suspect that it may be a contributing risk factor. Marijuana is the most commonly used recreational drug in the United States, according to the 2016 National Survey on Drug Use and Health [15]. More than 10% of the population reported its monthly usage in a 2017 survey [16]. In addition, the legalization of marijuana in some of the states in the US has increased its usage prevalence further, with every one in 10 people who use marijuana becoming addicted to it [16,17].

Several studies have identified smoking as a risk factor for delayed postoperative gastric leaks after LSG. For instance, Gluszynska et al. included 610 patients who underwent LSG and studied the risk factors for short-term and long-term postoperative complications [18]. The authors reported that nicotine is one of the independent risk factors associated with both short-term and long-term complications [18].

Very few studies are available describing the relation between marijuana use and post-LSG complications. Shockcor et al. studied 71 marijuana users and 71 control patients who underwent bariatric surgery [15]. The authors described that the patients in the marijuana group had a lower rate of long-term follow-up relative to the control group. The study also reported no statistically significant difference in the 30-day outcome between the cases and controls regarding the infection, bleeding, readmission, and reoperation rates [15]. However, the direct impact of marijuana on delayed gastric leaks is less clear. Further studies with a larger patient population are required to study the LSG complications secondary to marijuana use.

The management of gastric leak depends on the hemodynamic status of the patient. Patients with high clinical suspicion of gastric leak and hemodynamic instability must undergo surgical exploratory laparotomy despite negative imaging findings [11]. The management of clinically stable patients depends on the timing of gastric leak. Immediate surgical intervention with lavage and drainage with or without oversewing is recommended for leaks during the postoperative day (POD) 0-4 [11]. For leaks at >POD 4, the patients can be managed conservatively with intravenous hydration, proton pump inhibitors, broad-spectrum antibiotics, and imaging as required [11]. Surgical management can be considered if there is no clinical improvement or clinical deterioration [11].

## Conclusions

Through this case, we would like to highlight the potential association of marijuana consumption as one of the risk factors for delayed gastric leak post-LSG. While the mechanism is unknown, marijuana reduces the gastrointestinal motility and affects tissue healing. Hence, extensive preoperative assessment for substance abuse is recommended to prevent the post-LSG complications. Further studies with larger population is recommended to strongly establish the association between marijuana and bariatric complications.
